# *Belnapia mucosa* sp. nov. and *Belnapia arida* sp. nov., isolated from desert biocrust

**DOI:** 10.1099/ijsem.0.004837

**Published:** 2021-07-22

**Authors:** Esther Molina-Menor, Àngela Vidal-Verdú, Leila Satari, Alba Calonge-García, Javier Pascual, Juli Peretó, Manuel Porcar

**Affiliations:** ^1^​ Institute for Integrative Systems Biology I2SysBio (Universitat de València-CSIC), Calle del Catedràtic Agustin Escardino Benlloch 9, 46980 Paterna, Spain; ^2^​ Darwin Bioprospecting Excellence SL. Parc Científic Universitat de València, Calle del Catedràtic Agustin Escardino Benlloch 9, 46980 Paterna, Spain; ^3^​ Departament de Bioquimica i Biologia Molecular, Universitat de València, Calle del Dr. Moliner 5046100 Burjassot, Spain

**Keywords:** *Belnapia*, Tabernas Desert, biocrust, novel species, *Alphaproteobacteria*

## Abstract

Two novel Gram-staining-negative, aerobic, cocci-shaped, non-motile, non-spore forming, pink-pigmented bacteria designated strains T6^T^ and T18^T^, were isolated from a biocrust (biological soil crust) sample from the vicinity of the Tabernas Desert (Spain). Both strains were catalase-positive and oxidase-negative, and grew under mesophilic, neutrophilic and non-halophilic conditions. According to the 16S rRNA gene sequences, strains T6^T^ and T18^T^ showed similarities with *Belnapia rosea* CGMCC 1.10758^T^ and *Belnapia moabensis* CP2C^T^ (98.11 and 98.55% gene sequence similarity, respectively). The DNA G+C content was 69.80 and 68.96% for strains T6^T^ and T18^T^, respectively; the average nucleotide identity by blast (ANIb) and digital DNA–DNA hybridization (dDDH) values confirmed their adscription to two novel species within the genus *Belnapia*. The predominant fatty acids were summed feature 8 (C_18 : 1_ω7*c/*C_18 : 1_ω6*c*), C_16 : 0_, C_18 : 1_ 2-OH and summed feature 3 (C_16 : 1_ω7*c/*C_16 : 1_ω6*c*). According to he results of the polyphasic study, strains T6^T^ and T18^T^ represent two novel species in the genus *Belnapia* (which currently includes only three species), for which names *Belnapia mucosa* sp. nov. (type strain T6^T^ = CECT 30228^T^=DSM 112073^T^) and *Belnapia arida* sp. nov. (type strain T18^T^=CECT 30229^T^=DSM 112074^T^) are proposed, respectively.

The genus *Belnapia* was first described by Reddy *et al*. [1] and it is, at the time of writing, comprised of three species, which were all isolated from soil samples: *Belnapia moabensis* [1], *Belnapia rosea* [2] and *Belnapia soli* [3]. In this study we describe the polyphasic characterization of two strains, namely T6^T^ and T18^T^, which were isolated from biocrust (biological soil crust) samples from south-eastern Spain during a study on the microbial diversity of European arid regions.

Strains T6^T^ and T18^T^ were isolated in the vicinity of the Tabernas Desert (Almería, Spain) during a study on the culturable microbial diversity in European drylands [4]. The Tabernas Desert is considered the only arid desert in Europe. Biocrust samples were obtained from near the Tabernas Desert Parc (37.002404° N, 2.450655° W) and homogenized in phosphate buffered saline (PBS; NaCl 8.0 g l^−1^, KCl 0.2 g l^−1^, Na_2_HPO_4_ 1.44 g l^−1^, KH_2_PO_4_ 0.24 g l^−1^) pH 7.4 (1 g in 1 ml). The suspensions were then spread on 1, 0.1 and 0.01× trypticase soy agar (TSA; 15 g l^−1^ tryptone, 5 g l^−1^ NaCl, 5 g l^−1^ soya peptone), and Reasoner’s 2A Agar (R2A; 1 g l^−1^ peptone, 0.5 g l^−1^ yeast extract, 0.5 g l^−1^ dextrose, 0.5 g l^−1^ soluble starch, 0.3 g l^−1^ dipotassium phosphate, 0.05 g l^−1^ magnesium sulphate heptahydrate, 0.3 g l^−1^ sodium pyruvate). Agar was autoclaved separately and added before plating at a final concentration of 15 g l^−1^. The plates were incubated at 23 °C for 1 week. Strain T6^T^ was isolated from 0.1× TSA plates, whereas T18^T^ was isolated from a 0.01× TSA plate. The isolation of the strains was carried out by re-streaking on fresh media until a pure culture was obtained. Cell suspensions in TSA and R2A were cryopreserved at −80 °C with 15% glycerol (v/v). Their taxonomic status was determined by a polyphasic approach. On the basis of the results from phylogenetic, phenotypic and chemotaxonomic analysis, it is concluded that strains T6^T^ and T18^T^ are related to members of the genus *Belnapia* and representatives of two novel species. In the present work, the reference strains *B. rosea* DSM 23312^T^, *B. moabensis* DSM 16746^T^ and *B. soli* DSM 28067^T^, from the DSMZ (German Collection of Microorganisms and Cell Cultures, Leibniz Institute, Braunschweig, Germany), and strains T6^T^ and T18^T^ were all grown in parallel on R2A media at 30 °C, unless otherwise specified.

The phenotypic characteristics of T6^T^ and T18^T^ were analysed after 1 week of growth at 30 °C. A Gram staining test was carried out with KOH 3 % (w/v), recording viscosity as a negative result. Oxidase activity was tested by using the commercial Oxidase Test Stick for microbiology (PanReac AppliChem). Catalase activity was tested with hydrogen peroxide 30% (v/v), recording bubble formation as a positive result. Cell morphology was observed under an optical microscope with crystal violet glass stain. Growth at different temperatures (4, 10, 15, 20, 23, 30, 37, 40, 42 and 45 °C) and NaCl concentrations (0.0–4.0% at intervals of 0.5%) was checked on R2A. Growth at different pH values (4.0–10.0 at intervals of 1.0 pH unit) was examined by growing the strains in liquid R2A using the buffers MES (pH 4–6), HEPES (pH 7–8) and CHES (pH 9–10) at a final concentration of 10 mM. Growth under microaerophilic and anaerobic conditions was tested by incubating the plates in a candle jar and with the BD GasPak EZ pouch system (Becton, Dickinson), respectively. Carbon source assimilation and enzymatic activities were checked using the API 20NE and API ZYM system strips (bioMérieux) according to manufacturer’s instructions. BIOLOG GEN III MicroPlates (BIOLOG) were also used to determine carbon source assimilation.

Strains T6^T^, T18^T^ and the reference strains *B. rosea* DSM 23312^T^, *B. moabensis* DSM 16746^T^ and *B. soli* DSM 28067^T^ were grown on R2A medium at 30 °C for 72 h for analysis of cellular fatty acids. The analysis was carried out following the protocol recommended by MIDI Microbial Identification System (version 6.1, MIDI, Inc, Newark, DE, USA) [5]. The fatty acids content was analysed on a 6850 gas chromatography system (Agilent) using the TSBA6 method [6].

Genomic DNA extraction was carried out using the DNeasy Power Soil kit (Qiagen), according to the manufacturer’s instructions, but incubating at 65 °C after the addition of C1. Whole 16S rRNA gene PCR was carried out with universal primers 8F (5′-AGAGTTTGATCCTGGCTCAG-3′) [7] and 1492R (5′-GGTTACCTTGTTACGACTT-3′) [8] following procedures described previously [4]. Phylogenetic trees based on the 16S rRNA gene sequences were reconstructed using the maximum-likelihood (ML) [9] and neighbour-joining (NJ) [10] methods with the software mega X v.10.1.7. The TamuraNei G+I evolutionary model and the Kimura two-parameter model were used for the ML and NJ trees, respectively. The reliability of the branch patterns was assessed using bootstrap analysis based on 500 and 1000 replicates, respectively, for the ML and the NJ trees [11].

The draft genome of strains T6^T^ and T18^T^ were sequenced with the NovaSeq 6000 system (Illumina; 2×150 bp paired-end sequencing). The genomic DNA was randomly fragmented by sonication, then DNA fragments were end polished, A-tailed and ligated with the full-length adapters for Illumina sequencing. Further PCR amplification was carried out with P5 and indexed P7 oligonucleotides, and PCR products for the final construction of the libraries were purified with an AMPure XP system. Libraries were then checked for size distribution by Agilent 2100 Bioanalyzer (Agilent Technologies), and quantified by real-time PCR. The FastQC tool (v0.11.5) [12] was utilized to assess the quality of the sequence reads. There were 14 375 848 and 12 879 132 paired-end reads for strains T6^T^ and T18^T^, respectively before filtering. After quality filtering, there were considered to be 13 106 155 and 11 792 897 paired-end sequences the genomes of strains T6^T^ and T18^T^, respectively. Genome assembly of paired reads was performed using the ‘--isolate’ mode in SPAdes (3.14.1) [13]. Assembly statistics were calculated with QUAST (v.5.0.2) [14] and the completeness and contamination levels were evaluated with CheckM (v.1.1.3) [15]. The draft genomes were annotated using the RAST tool kit (RAStk) [16] integrated in PATRIC v.3.6.8. The draft genomes were analysed with the TYGS tool [17] in order to identify the most closely related type strains to T6^T^ and T18^T^ with publicly available genomes and to calculate digital DNA–DNA hybridization (dDDH) indexes. JSpecies [18] was used for calculating the average nucleotide identities according to blast (ANIb) between genome pairs. UBCG (v.3.0) [19] was used for reconstructing the phylogenomic tree among the selected strains based on a multiple alignment of a set of 92 housekeeping genes. We selected the alignment method codon and inferred the phylogenetic relationships with FastTree. The reliability of the branch patterns was assessed using bootstrap analysis based on 100 replicates.

Strains T6^T^ and T18^T^ were aerobic, Gram-staining-negative, non-motile and coccus-shaped (0.8–1.0 µm in diameter). The cells of both strains occurred singly, as in other members of the genus *Belnapia*. Colonies were pink, irregular and mucous. T6^T^ colonies were paler than those of the rest of the members of the genus *Belnapia*. After 3–5 days of growth at 30 °C, the colonies of both strains displayed a diameter of around 3–4 mm.

Both strains were able to grow at between 4 and 40 °C (optimum at 30 °C). Moreover, T6^T^ was able to grow at up to 42 °C. Both strains showed tolerance to up to 1.5% (w/v) of NaCl (optimum 0–1%). *B. moabensis* DSM 16746^T^ and *B. rosea* DSM 23312^T^ showed similar NaCl tolerances, in contrast with *B. soli* DSM 28067^T^, which was able to grow at concentrations of up to 3%. All five strains were able to grow at between pH 5 and 9, with an optimum at 6–7 (Table 1).

**Table 1.** Differential phenotypic characteristics between strains T6^T^, T18^T^ and the other members of the genus *Belnapia* Strains: 1, T6^T^; 2, T18^T^; 3, *Belnapia moabensis* DSM 16746^T^; 4, *Belnapia rosea* DSM 23312^T^; 5, *Belnapia soli* DSM 28067^T^. Data for reference strains were obtained in the present study. +, Positive; −, negative; w, weak reaction. All strains are positive for alkaline phosphatase, esterase (C4), esterase lipase (C8), leucine arylamidase, acid phosphatase and napthol-AS-BI-phosphohydrolase. All strains are negative for lipase (C14), valine arylamidase, cystine arylamidase, α-chymotrypsin, α-galactosidase, β-galactosidase, β-glucuronidase, α-glucosidase, β-glucosidase, *N*-acetyl-β-glucosaminidase, α-mannosidase, α-fucosidase, fermentation of glucose, arginine dihydrolysis, aesculin hydrolysis and the assimilation of d-mannitol, *N*-acetyl-glucosamine, maltose, capric acid, trisodium citrate and phenylacetic acid.

Characteristic	1	2	3	4	5
Isolation source	Biocrust	Biocrust	Soil crust	Forest soil	Grass soil
**Growth at/in**					
Temperature range (°C)	4–42	4–40	4–40	4–40	4–40
pH range	5–9	5–9	5–9	5–9	5–9
NaCl tolerance (%, w/v)	0–1.5	0–1.5	0–2	0–1.5	0–3
**Carbon source utilization (API 20NE**)					
d-glucose	−	−	−	−	w
l-arabinose	−	−	w	w	w
d-mannose	−	−	−	w	−
Potassium gluconate	w	−	−	w	−
Adipic acid	w	−	−	+	+
Malic acid	w	−	−	−	w
**Enzymatic activity (API 20NE**)					
Nitrate reduction	−	−	−	+	−
Indole production	−	−	−	+	−
Urease	−	w	+	w	w
Gelatin	−	−	+	−	−
**Enzymatic activity (API ZYM**)					
Trypsin	−	−	+	−	−

Strains T6^T^ and T18^T^ showed, like the other members of the genus *Belnapia*, a positive response for alkaline phosphatase, esterase (C4), esterase lipase (C8), leucine arylamidase, acid phosphatase and napthol-AS-BI-phosphohydrolase. In contrast, T6^T^ and T18^T^ and their relatives of the genus *Belnapia* showed a negative response for lipase (C14), valine arylamidase, cystine arylamidase, α-chymotripsin, α-galactosidase, β-galactosidase, β-glucuronidase, α-glucosidase, β-glucosidase, *N*-acetyl-β-glucosaminidase, α-manosidase, α-fucosidase, fermentation of glucose, arginine dihydrolysis and aesculin hydrolysis. *B. moabensis* DSM 16746^T^ showed positive response for trypsin and gelatin, while *B. rosea* DSM 23312^T^ showed positive response for reduction of nitrate and indole production. T6^T^ was negative for urease, in contrast to the other four strains, which were positive or weakly positive for this activity. In API 20 NE strips, T18^T^ was not able to assimilate any of the saccharides tested, whereas T6^T^ could grow weakly with potassium gluconate, adipic acid and malic acid (Table 1). Furthermore, the results of the BIOLOG GENIII plates indicated that strains T6^T^ and T18^T^ were only able to oxidize five and four out of the 71 carbon sources, respectively (Table S1, available in the online version of this article). In contrast, the reference strains *B. moabensis* DSM 16746^T^, *B. rosea* DSM 23312^T^ and *B. soli* DSM 28067^T^ were able to oxidize 41, 25 and 16 carbon sources, respectively. This indicates that the reference strains present a more polytrophic metabolism than strains T6^T^ and T18^T^.

Almost complete 16S rRNA gene sequences were obtained. The 16S rRNA gene sequence lengths of strains T6^T^ and T18^T^ are 1392 (accession number MW583035) and 1383 bp (MW583036), respectively. According to the EzBioCloud database tool, the most closely related type strains of T6^T^ were *B. rosea* CGMCC 1.10758^T^ (98.11%), *B. moabensis* CP2C^T^ (97.38%) and *B. soli* PB-K8^T^ (96.80%); whereas the closest relatives of T18^T^ were *B. moabensis* CP2C^T^ (98.55%), *B. soli* PB-K8^T^ (97.54%) and *B. rosea* CGMCC 1.10758^T^ (97.40%). The type strains *B. rosea* DSM 23312^T^, *B. moabensis* DSM 16746^T^ and *B. soli* DSM 28067^T^ were, thus, selected as comparative reference strains, which were obtained from the DSMZ-German Collection of Microorganisms and Cell Cultures (Leibniz Institute, Braunschweig, Germany).

The phylogenetic positions of strains T6^T^ and T18^T^ within the genus *Belnapia* were confirmed by both 16S-rRNA-based ML and NJ phylogenetic tress (Figs 1 and S1). T6^T^ grouped with *B. rosea* CGMCC 1.10758^T^, whereas T18^T^ showed an external position in the cluster formed by *Belnapia moabensis* CP2C^T^ and *Belnapia soli* PB-K8^T^ in both trees. This phylogenetic inference was supported by high bootstrap values.

**Fig. 1.**
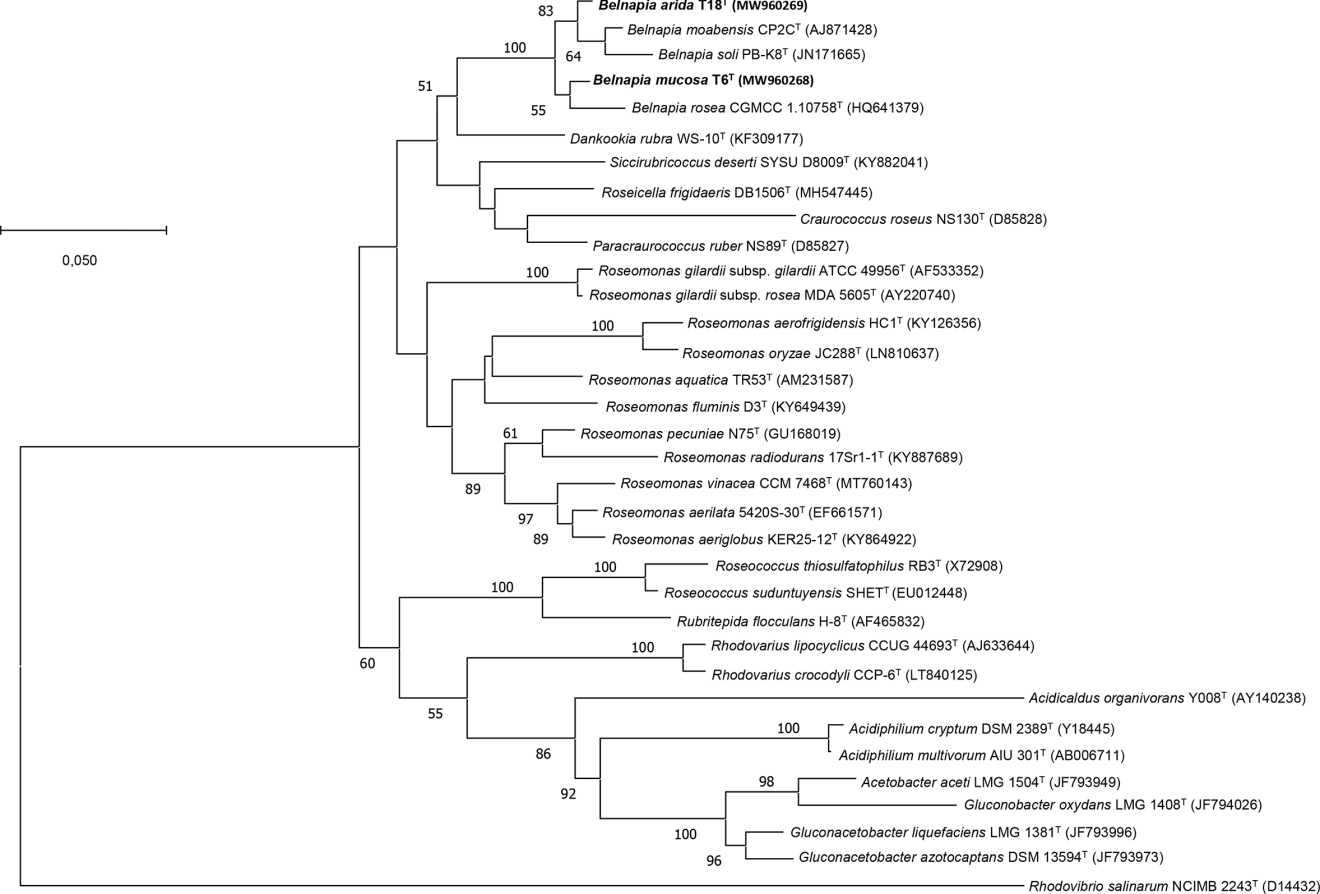
Maximum likelihood phylogenetic tree showing the relationships between strains T6^T^, T18^T^ and other members of the family *Acetobacteraceae* based on 16S rRNA gene sequences. The optimal evolutionary model of nucleotide substitution applied was Tamura–Nei G+I. Numbers at branch points refer to bootstrap percentages based on 500 replicates (values under 50% are not indicated). *Rhodovibrio salinarum* NCIMB 2243^T^ (D14432) was used as an outgroup. Bar, 0.05 fixed nucleotide substitutions per site.

The draft genomes of strains T6^T^ and T18^T^ consisted of 220 and 355 contigs, respectively, which constituted a total length of 6449681 and 6937094 bp, respectively. The N50 values were 328 210 and 194 160 for T6^T^ and T18^T^ respectively. The genomic DNA G+C contents were 69.80 and 68.96% for T6^T^ and T18^T^ respectively, which is in accordance with the values previously described for the rest of the species within the genus *Belnapia* and further confirms their adscription to this genus [1–3]. A total of 6369 and 7450 coding sequences (CDSs) were predicted for strains T6^T^ and T18^T^, of which 3380 and 3480, respectively, corresponded to proteins with functional assignment. Regarding the prediction of tRNA and rRNAs, a total of 49 and 47 tRNAs, and 3 and 2 rRNAs were predicted for strains T6^T^ and T18^T^, respectively. The 16S rRNA gene sequences of strains T6^T^ and T18^T^ were also extracted from the genome, which were 1496 and 1494 bp long, respectively (accession numbers MW960268 and MW960269, respectively). According to the EzBioCloud database tool, the most closesly related type strains to T6^T^ were *B. rosea* CGMCC 1.10758^T^ (98.16%), *B. moabensis* CP2C^T^ (97.45%) and *B. soli* PB-K8^T^ (96.80%); whereas the most closely related type strains to T18^T^ were *B. moabensis* CP2C^T^ (98.58%), *B. soli* PB-K8^T^ (97.54%) and *B. rosea* CGMCC 1.10758^T^ (97.45%). The completeness values of genomes were 100 and 99.5 % for strains T6^T^ and T18^T^, respectively; and the levels of contamination were 0.5 and 2.49%, respectively. The draft genomes showed, thus, sufficient quality for further analysis.

In order to obtain a more accurate phylogenetic inference of novel strains, a phylogenomic tree based on nucleotide sequences was reconstructed (Fig. 2). The phylogenomic tree corroborated that the two strains represent members of the genus *Belnapia*. Strain T18^T^ was most closely related to *B. moabensis* DSM 16746T, while T6^T^ showed an external position to the rest of the members of the genus *Belnapia*. The type strain of *B. soli* was not included in this analysis because its genome was not publicly available at the time of writing.

**Fig. 2.**
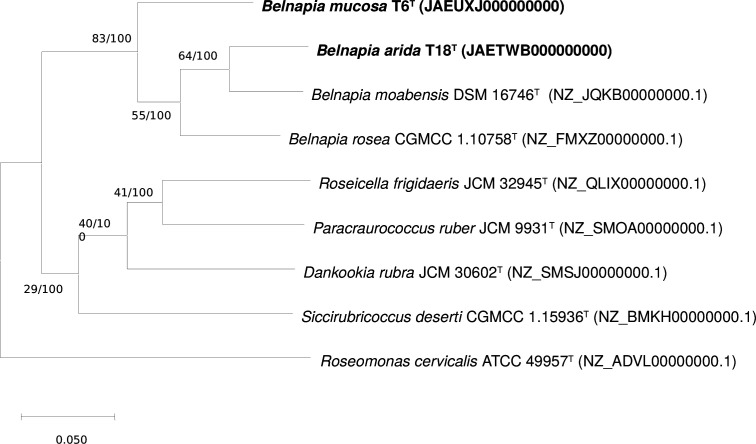
Phylogenomic tree showing the relationships between strains T6^T^, T18^T^ and other members of the family *Acetobacteraceae* based on a multiple alignment of a set of 92 gene sequences using the UBCG (v.3.0) [19] pipeline. Bootstrap analysis was carried out using 100 replicates. Gene support indices (maximum value; 92 genes) and percentage bootstrap values (maximum value; 100%) are given at the branch points. *Roseomonas cervicalis* ATCC 49957^T^ was used as an outgroup. Bar 0.050 substitutions per position.

The ANIb and digital DDH values between strains T6^T^ and T18^T^ and other related species were calculated (Table S2). The ANIb and dDDH values of strain T6^T^ vs. *Belnapia rosea* CGMCC 110758^T^ were 83.26 and 29 %, respectively; the ANIb and dDDH values of strain T18^T^ vs. *Belnapia moabensis* DSM 16746^T^ were 88.47 and 40.5 %, respectively. Moreover, both genome indexes were calculated between strains T6^T^ and T18^T^, which were 82.96 and 28.5 % for ANIb and dDDH, respectively. As the values were below the thresholds established to circumscribe prokaryotic species, namely 95 % for ANI values [20] and 70 % for dDDH [21], both genome indexes confirmed the classification of strains T6^T^ and T18^T^ as representing novel species [22].

The analysis of the genome of strains T6^T^ and T18^T^ allowed the prediction of their ability to synthesize phosphatidylglycerol, phosphatidylcholine and diphosphatidylglycerol on the basis of the presence of genes coding for phosphatidylglycerol phosphatase (EC 3.1.3.27), phosphatidylethanolamine *N*-methyltransferase (EC 2.1.1.17) and cardiolipin synthase (EC 2.7.8.-) respectively. This polar lipids’ profile is in agreement with the polar lipid analyses results available for other species of the genus *Belnapia* [1–3]. Furthermore, their ability to synthesize phosphatidylethanolamine was predicted on the basis of the presence of the genes coding for phosphatidylserine synthase (EC 2.7.8.8) and phosphatidylserine decarboxylase (EC 4.1.1.65), which had been previously described for *B. soli* [3].

The major fatty acid for strains T6^T^ and T18^T^ was summed feature 8 (C_18 : 1_ω7*c/*C_18 : 1_ω6*c*) (41.4 and 51.5%, respectively). However, there were also high amounts of C_16 : 0_ (15.7 and 12.9%, respectively for T6^T^ and T18^T^), C_18 : 1_ 2-OH (12.2 and 10.0%, respectively) and summed feature 3 (C_16 : 1_ω7*c/*C_16 : 1_ω6*c*) (10.3 and 12.2%, respectively) (Table 2). This is in accordance with the profiles obtained for the members of the genus *Belnapia*, which also showed high amounts of summed feature 8, summed feature 3, C_18 : 1_ 2-OH and C_16 : 0_, thus confirming the inclusion of both strains within the genus *Belnapia*.

**Table 2.** Cellular fatty acid composition (percentages) of strains T6^T^, T18^T^ and the members of the genus *Belnapia* Strains: 1, T6^T^; 2, T18^T^; 3, *Belnapia moabensis* DSM 16746^T^; 4, *Belnapia rosea* DSM 23312^T^; 5, *Belnapia soli* DSM 28067^T^. tr, trace (<1.0%); –, not detected.

	1	2	3	4	5
**Saturated**					
C_9 : 0_	tr	tr	–	tr	tr
C_10 : 0_	–	tr	–	tr	tr
C_14 : 0_	1.9	tr	–	tr	1.2
C_16 : 0_	15.7	12.9	13.2	11.3	16.8
C_18 : 0_	3.6	1.8	1.9	1.2	2.6
C_19 : 0_cyclo ω8*c*	–	–	–	1.8	2.3
**Unsaturated**					
C_16 : 1_ω5*c*	–	tr	–	1.0	tr
C_18 : 1_ω5*c*	–	–	–	tr	–
C_18 : 1_ω9*c*	5.4	3.5	1.7	1.2	2.4
**Hydroxylated**					
C_12 : 0_ 2-OH	6.4	3.9	2.0	1.6	2.3
C_16 : 0_ 3-OH	–	tr	–	tr	1.4
C_18 : 1_ 2-OH	12.2	10.0	8.2	9.0	10.7
C_18 : 0_ 3-OH	1.4	–	–	–	tr
Summed Features*					
Summed feature 2	1.1	tr	tr	tr	1.3
Summed feature 3	10.3	12.2	10.2	19.2	16.5
Summed feature 8	41.4	51.5	62.0	50.6	40.4

*Summed features are fatty acids that cannot be resolved reliably from another fatty acid using the chromatographic conditions chosen. The MIDI system groups these fatty acids together as one feature with a single percentage of the total. Summed feature 2 corresponds to C_14 : 0_ 3-OH/iso-C_16 : 1_I/C_12 : 0_ aldehyde; summed feature 3 corresponds to C_16 : 1_ω7*c/*C_16 : 1_ω6*c*; summed feature 8 corresponds to C_18 : 1_ω7*c/*C_18 : 1_ω6*c*.

The results of the phenotypic, chemotaxonomic, genomic and phylogenetic analyses confirm that strains T6^T^ and T18^T^ should be considered as each representing a novel species within the genus *Belnapia*, for which the names *Belnapia mucosa* sp. nov. and *Belnapia arida* sp. nov., respectively, are proposed.

## Description of *Belnapia mucosa* sp. nov.

*Belnapia mucosa* (mu.co’sa. L. fem. adj. *mucosa*, mucous, slimy).

Colonies are circular, smooth, mucous, convex and pale-pink. Cells are Gram-reaction-negative, coccoid (0.8–1.0 µm) and non-motile. Growth occurs at 4–42 °C (optimum at 30 °C) and pH 5–9 (optimum 6–7), and it can tolerate up to 1.5 % (w/v) NaCl (optimum 0–1%). This species grows under aerobic and microaerophilic conditions, no growth is observed under anaerobic conditions. Alkaline phosphatase, esterase (C4), esterase lipase (C8), leucine arylamidase, acid phosphatase and naphthol-AS-BI-phosphohydrolase activities are detected. Lipase (C14), valine arylamidase, cystine arylamidase, trypsin, α-chymotrypsin, α-galactosidase, β-galactosidase, β-glucuronidase, α-glucosidase, β-glucosidase, *N*-acetyl-β-glucosaminidase, α-mannosidase, α-fucosidase, nitrate reduction, indole production, glucose fermentation, arginine dihydrolysis, urease, aesculin hydrolysis and gelatinase are not detected. According to API 20 NE test kits, this species is weakly positive for the assimilation of potassium gluconate, adipic acid and malic acid and negative for the assimilation of d-glucose, l-arabinose, d-mannose, d-mannitol, *N*-acetyl-glucosamine, maltose, capric acid, trisodium citrate and phenylacetic acid. According to BIOLOG GENIII MicroPlates, this species is positive for the utilization of l-galactonic acid lactone, β-hydroxy-dl-butyric acid, d-glucuronic acid, glucuronamide and acetoacetic acid; and negative for raffinose, α-d-glucose, d-sorbitol, gelatin, pectin, *p*-hydroxy-phenylacetic acid, Tween 40, dextrin, lactose, d-mannose, d-mannitol, glycyl-l-proline, d-galacturonic acid, methyl pyruvate, γ-aminobutyric acid, maltose, melibiose, d-fructose, d-arabitol, l-alanine, d-lactic acid methyl ester, α-hydroxybutyric acid, trehalose, methyl β-d-glucoside, d-galactose, *myo*-inositol, l-arginine, d-gluconic acid, l-lactic acid, cellobiose, d-salicin, 3-methyl-d-glucoside, glycerol, l-aspartic acid, citric acid, α-ketobutyric acid, gentiobiose, *N*-acetyl-d-glucosamine, d-fucose, d-glucose-6-phosphate, l-glutamic acid, α-ketoglutaric acid, sucrose, *N*-acetyl-β-d-mannosamine, l-fucose, d-fructose-6-phosphate, l-histidine, mucic acid, d-malic acid, propionic acid, turanose, *N*-acetyl-d-galactosamine, l-rhamnose, d-aspartic acid, l-pyroglutamic acid, quinic acid, l-malic acid, acetic acid, stachyose, *N*-acetyl neuraminic acid, inosine, d-serine, l-serine, d-saccharic acid, bromo-succinic acid and formic acid. The major fatty acids are summed feature 8 (C_18 : 1_ω7*c/*C_18 : 1_ω6*c*), C_16 : 0_, C_18 : 1_ 2-OH and summed feature 3 (C_16 : 1_ω7*c/*C1_6 : 1_ω6*c*).

The type strain T6^T^ (CECT 30228^T^=DSM 112073^T^) was first isolated from the Tabernas Desert in Almería (Spain) from a biocrust sample. The DNA G+C content of the type strain is 69.80%. The DDBJ/ENA/GenBank accession number for the 16S rRNA gene sequence is MW960268 and the genome accession number is JAEUXJ000000000.

## Description of *Belnapia arida* sp. nov.

*Belnapia arida* (a’ri.da. L. fem. adj. *arida*, dry, referring to the isolation of the strain from an arid soil)

Colonies are circular, smooth, mucous, convex and pink. Cells are Gram-reaction-negative, coccoid-shaped (0.8–1.0 µm) and non-motile. Growth occurs at 4–40 °C (optimum at 30 °C) and pH 5–9 (optimum 6–7), and it can tolerate up to 1.5% (w/v) NaCl (optimum 0–1%). This species grows under aerobic and microaerophilic conditions, no growth is observed under anaerobic conditions. Alkaline phosphatase, esterase (C4), esterase lipase (C8), leucine arylamidase, acid phosphatase, naphthol-AS-BI-phosphohydrolase and urease activities are detected. Lipase (C14), valine arylamidase, cystine arylamidase, trypsin, α-chymotrypsin, α-galactosidase, β-galactosidase, β-glucuronidase, α-glucosidase, β-glucosidase, *N*-acetyl-β-glucosaminidase, α-mannosidase, α-fucosidase, nitrate reduction, indole production, glucose fermentation, arginine dihydrolysis, aesculin hydrolysis and gelatinase are not detected. According to API 20 NE test kits, this species is negative for the assimilation of d-glucose, l-arabinose, d-mannose, d-mannitol, *N*-acetyl-glucosamine, maltose, potassium gluconate, capric acid, adipic acid, malic acid, trisodium citrate and phenylacetic acid. According to BIOLOG GENIII MicroPlates, this species is positive for the utilization of d-gluconic acid, β-hydroxy-dl-butyric acid, l-pyroglutamic acid and l-malic acid; and negative for the utilization of raffinose, α-d-glucose, d-sorbitol, gelatin, pectin, *p*-hydroxyphenylacetic acid, Tween 40, dextrin, lactose, d-mannose, d-mannitol, glycyl-l-proline, d-galacturonic acid, methyl pyruvate, γ-aminobutyric acid, maltose, melibiose, d-fructose, d-arabitol, l-alanine, l-galactonic acid lactone, d-lactic acid methyl ester, α-hydroxybutyric acid, trehalose, methyl β-d-glucoside, d-galactose, *myo*-inositol, l-arginine, l-lactic acid, cellobiose, d-salicin, 3-methyl-d-glucoside, glycerol, l-aspartic acid, d-glucuronic acid, citric acid, α-ketobutyric acid, gentiobiose, *N*-acetyl-d-glucosamine, d-fucose, d-glucose-6-phosphate, l-glutamic acid, glucuronamide, α-ketoglutaric acid, acetoacetic acid, sucrose, *N*-acetyl-β-d-mannosamine, l-fucose, d-fructose-6-phosphate, l-histidine, mucic acid, d-malic acid, propionic acid, turanose, *N*-acetyl-d-galactosamine, l-rhamnose, d-aspartic acid, quinic acid, acetic acid, stachyose, *N*-acetyl neuraminic acid, inosine, d-serine, l-serine, d-saccharic acid, bromosuccinic acid and formic acid. The major fatty acids are summed feature 8 (C_18 : 1_ω7*c/*C_18 : 1_ω6*c*), C_16 : 0_, summed feature 3 (C_16 : 1_ω7*c/*C_16 : 1_ω6*c*) and C_18 : 1_ 2-OH.

The type strain T18^T^ (CECT 30229^T^=DSM 112074^T^) was first isolated from the Tabernas Desert in Almería (Spain) from a biocrust sample. The DNA G+C content of the type strain is 68.96%. The DDBJ/ENA/GenBank accession number for the 16S rRNA gene sequence is MW960269 and the genome accession number is JAETWB000000000.

## Supplementary Data

Supplementary material 1
