# Supplementary material for: Belnapia mucosa sp. nov. and Belnapia arida sp. nov., isolated from desert biocrust
Source: Int J Syst Evol Microbiol. 2021 Jul 22;71(7):004837. doi: 10.1099/ijsem.0.004837 (PMC8489839; doi:10.1099/ijsem.0.004837)
Supplement: Supplementary material 1 [file ijsem-71-4837-s001.pdf]

Belnapia sp. nov. and Belnapia sp. nov., isolated from the Tabernas Desert (Almería, Spain)

Esther Molina-Menor[1], Àngela Vidal-Verdú[1], Leila Satari[1], Alba Calonge-García[1], Javier Pascual[2], Juli Peretó[1,2,3] and Manuel Porcar[1,2]

ORCiD: EMM 0000-0002-8604-5052; AVV 0000-0001-7923-4835; LS 0000-0001-6795-8254; ACG 0000-0003-4146-0655; JPa 0000-0002-1900-7099; JPe 0000-0002-5756-1517; MP 0000-0002-7916-9479

1. Institute for Integrative Systems Biology I2SysBio (Universitat de València-CSIC), Calle del Catedràtic Agustin Escardino Benlloch 9, 46980 Paterna, Spain
2. Darwin Bioprospecting Excellence SL. Parc Científic Universitat de València, Calle del Catedràtic Agustin Escardino Benlloch 9, 46980 Paterna, Spain
3. Departament de Bioquimica i Biologia Molecular, Universitat de València, Calle del Dr. Moliner 50, 46100 Burjassot, Spain

***** Correspondence: manuel.porcar@csic.es

Keyword

Belnapia, Tabernas Desert, biocrust, new species, Alphaproteobacteria

Repositories

The 16S rRNA gene sequences of strains T6^T and T18^T have been deposited in DDBJ/ENA/GenBank under the accession numbers MW960268 and MW960269, respectively. The genomic sequences of strains T6^T and T18^T have been deposited under the DDBJ/ENA/GenBank accession numbers JAEUXJ000000000 and JAETWB000000000, respectively

Supplementary Table 1. Carbon source utilization comparison using Gen III Micro Plates of strains T6[T], T18[T] and the type strains of closely related *Belnapia* species. Strains: 1, T6[T]; 2, T18[T]; 3, *Belnapia moabensis* DSM 16746[T]; 4, *Belnapia rosea* DSM 23312[T]; 5, *Belnapia soli* DSM 28067[T]. Data for reference strains were obtained in the present study. +, positive; -, negative; W, weak reaction. All strains are positive for β-Hydroxy-D,L-butyric acid. All strains are negative for D-raffinose, pectin, dextrin, α-D-lactose, D-mannose, D-mannitol, glycyl-L-proline, D-maltose, D-melibiose, D-trehalose, β-Methyl-D-glucoside, L-arginine, D-cellobiose, D-salicin, 3-methyl-D-glucoside, L-aspartic acid, gentiobiose, D-glucose-6PO4, N-acetyl-β-D-mannosamine, D-fructose-6PO4, L-histidine, L-rhamnose, inosine, D-serine and bromo-succinic acid.

**Characteristic**	**1**	**2**	**3**	**4**	**5**
α-D-Glucose	-	-	+	-	-
D-Sorbitol	-	-	+	-	-
Gelatin	-	-	+	-	-
p-Hydroxy-phenylacetic acid	-	-	+	-	-
Tween 40	-	-	-	+	-
D-Galacturonic acid	-	-	+	+	+
Methyl pyruvate	-	-	+	-	+
γ-Amino-butyric acid	-	-	-	+	-
D-Fructose	-	-	+	-	-
D-Arabitol	-	-	+	-	-
L-Alanine	-	-	+	+	-
L-Galactonic acid lactone	+	-	+	+	+
D-Lactic acid methyl ester	-	-	+	+	-
α-Hydroxy-butyric acid	-	-	+	-	+
D-Galactose	-	-	+	-	-
myo-Inositol	-	-	+	-	-
D-Gluconic acid	-	+	+	+	-
L-Lactic acid	-	-	+	+	+
Glycerol	-	-	+	-	+
D-Glucuronic acid	+	-	+	+	+
Citric acid	-	-	-	+	-
α-Keto-butyric acid	-	-	+	-	-
N-acetyl-D-glucosamine	-	-	+	-	-
D-Fucose	-	-	+	+	+
L-Glutamic acid	-	-	+	-	+
Glucuronamide	+	-	+	+	+
α-Keto-glutaric acid	-	-	+	+	+
Acetoacetic acid	+	-	+	+	-
Sucrose	-	-	+	-	-
L-Fucose	-	-	+	-	-
Mucic acid	-	-	+	+	+
D-Malic acid	-	-	+	+	+
Propionic acid	-	-	+	+	-
D-Turanose	-	-	+	-	-
N-acetyl-D-galactosamine	-	-	+	-	-
D-Aspartic acid	-	-	+	-	-
L-Pyroglutamic acid	-	+	+	+	-
Quinic acid	-	-	-	+	-
L-Malic acid	-	+	+	+	+
Acetic acid	-	-	+	+	-
Stachyose	-	-	+	-	-
N-Acetyl neuraminic acid	-	-	-	+	-
L-Serine	-	-	+	-	-
D-Saccharic acid	-	-	+	+	+
Formic acid	-	-	+	+	-

Supplementary Figure 1. Neighbour-Joining phylogenetic tree showing the relationships between strains T6^{T}, T18^{T} and other members of the family *Acetobacteraceae* based on 16S rRNA gene sequences. The evolutionary model of nucleotide substitution applied is Kimura two-parameter model (K2P). Numbers at branch points refer to bootstrap percentages based on 1000 replicates (values under 50% are not indicated). *Rhodovibrio salinarum* NCIMB 2243^{T} (D14432) was used as an outgroup. Bar 0.02 fixed nucleotide substitutions per site.

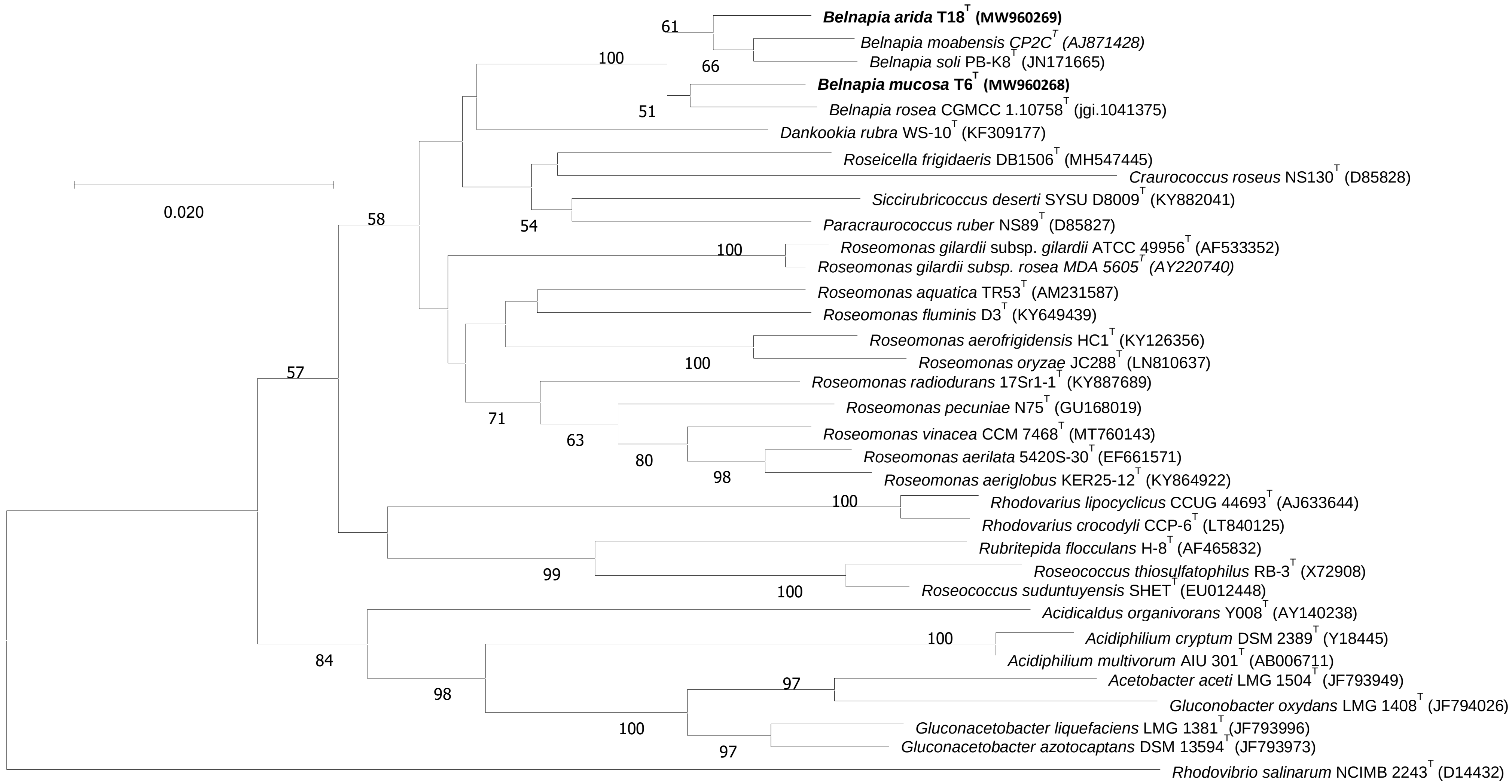

Supplementary Table 2. Genomic distance indexes for strains T6^T and T18^T, compared to other type strains of the genus *Belnapia*. Average Nucleotide Identity values (ANIb) and estimated digital DNA-DNA Hybridization (dDDH) values (%) are shown. 1.T6^T; 2. T18^T; 3. *B. moabensis* DSM 16746^T; 4. *B. rosea* CGMCC 110758^T.

**ANIb**	**1**	**2**	**3**	**4**
1- T6^T	*	83.07	82.86	83.26
2- T18^T	82.96	*	88.47	84.42
**Estimated dDDH**				
1- T6^T	*	28.5	28.3	29.0
2- T18^T	28.5	*	40.5	30.2
